# The agro-enabled urban revolution, pesticides, politics, and popular culture: a case study of land use, birds, and insecticides in the USA

**DOI:** 10.1007/s11356-019-05305-9

**Published:** 2019-05-26

**Authors:** Richard A. Brain, Julie C. Anderson

**Affiliations:** 1 Syngenta Crop Protection LLC, Greensboro, NC USA; 2 Richardson College for the Environment, The University of Winnipeg, Winnipeg, Manitoba Canada

**Keywords:** Land use, Breeding bird survey, Agriculture, Pesticides, Bird declines

## Abstract

Urbanization is an inevitable process in human civilization. When populations expand, socio-economic and political dynamics typically shift from agricultural predominance to one of industry and services. Accordingly, agrarian societies transform from diffuse rural communities to dense urban centers. By 2050, the world’s population is projected to reach 9.1 billion, with the urban population growing from 50 to 70%. Inevitably, this ever-expanding urban frontier encroaches along the human-ecological interface, creating a challenge for conservation and biodiversity. For the past 30 years, agricultural cropland area in the USA has remained fairly constant, despite significant population growth over the same time period. Thus, agricultural production in America has more than kept pace with rapid population growth and global export demand without increasing the farmland footprint at the expense of wildlife habitat. This is primarily due to considerable advances made in pesticide development, safety, and regulation, coupled with soil conservation and genetically modified crops. Still, the potential contribution of agriculture to ecosystem impairment remains contentious, particularly with regard to current use of pesticides. Recently, significant focus has been placed on the state of bird populations in the USA. Many species are considered imperiled, and this is often attributed in the popular media to pesticide use. However, focusing solely on the agricultural/chemical story as a significant driver of species viability and ecological risk within the broader biodiversity and conservation narrative lacks context and perspective. Moreover, the hypothesis that pesticides are indirectly affecting bird population status via reductions in food resources should be considered with caution and within the context of other likely causes. This work explores the dynamics between historical land use, human-controlled activity, and bird population trends from a holistic perspective within the USA. The aim is to provide context, developed from a relative comparison of potential contributing factors, in order to help inform discussion and foster dialogue between industry, academia, government, non-governmental organizations, and the public.

**Electronic supplementary material:**

The online version of this article (10.1007/s11356-019-05305-9) contains supplementary material, which is available to authorized users.

## Introduction

The pesticide industry requires regulation; a strong, science-based regulatory system is essential to ensuring that pesticides meet rigorous safety standards while also protecting crops and increasing agricultural outputs. Indelible historical examples of ecological naiveté (e.g., DDT, see Carson 1962) and inadequate recognition and consideration of human health impacts (e.g., Agent Orange, see Stellman et al. 2003) certainly exist. It is important to recognize the historical impact of these examples and to appreciate that a desire to avoid repeating these missteps was among the driving forces that culminated in the establishment of rigorous industry regulation and oversight manifested through the formation of the United States Environmental Protection Agency in 1970. Still, the fact that the pesticide industry is now intensively and stringently regulated under the Federal Insecticide, Fungicide, and Rodenticide Act (FIFRA) (CFR 2014) is both widely misunderstood and underappreciated. Compounding this issue is a tendency, either consciously or sub-consciously, towards the sensational, where research reporting adverse effects of pesticides is significantly more likely to be published in high-impact factor journals than that reporting no effects, if in fact research reporting no-effects is published at all (Hanson et al. 2018). Consequently, this synthetic bias towards the adverse can prevent the public from being knowledgeable participants in policy discussions about scientific issues (Ransohoff and Ransohoff 2001).

The USA has transitioned from a predominantly agrarian society, where more than half (54.4%) the population lived in rural landscapes in 1910, to an urban-dominated society a century later, with over 80% of the population now living in cities (USCB 2016). Globally, the United Nations predicts that the world’s urban population will grow by more than a billion people between 2010 and 2025, while no increase in rural population is projected (UN 2007). By 2050, the world’s population is expected to increase by 34% to 9.1 billion with the urban population growing from ~ 50% at present to 70% (FAO 2009). Moreover, dietary preferences are also changing to reflect an increased demand for more energy-intensive agricultural products (e.g., meat) as well as a propensity for more processed and pre-prepared foods (Popkin 2001; de Haen et al. 2003). Consequently, in order to meet this demand, it is estimated that food production will need to increase by 70%, with a 30% increase in annual cereal production (to 3 billion tonnes) and > 40% increase in annual meat production (to 470 million tonnes) (FAO 2009).

As Satterthwaite et al. (2010) concluded: “no nation has prospered without urbanization and there is no prosperous nation that is not predominantly urban.” Beginning in the mid-eighteenth century, agricultural societies became more industrialized and urban ushering in the age of industrial revolution. Following the Second World War, there was a dramatic socio-economic shift from agriculture to industry and services. Currently, ~ 97% of the world’s gross domestic product (GDP) is generated from, and 65% of the world’s economically active population works in, industry- or service-related sectors (Satterthwaite et al. 2010). In contrast, there are only about 3 million farmers and ranchers presently in the USA, comprising less than 1% of the population, but providing food for the remainder, with food exports exceeding imports (USDA 2012; USDA ERS 2017, 2018).

Not surprisingly, urban centers typically emerge proximal to highly fertile soils for the purposes of food production, but thereupon, urban expansion often occurs over the most productive agricultural land, ultimately exceeding the capacity for agricultural production of the surrounding area (Satterthwaite et al. 2010; Imhoff et al. 2004). Accordingly, densely concentrating population centers require intensive externalized food sources with complex global supply chains (Rees 1992). Thus, in order to sustain an expanding urban population, farmers ultimately need to produce greater yields from less land, given that further arable land development would be at the expense of habitat needed to support biodiversity.

Undeniably, despite these ensuing challenges from shifting population demographics, agriculture has more than kept pace with increasing global demand. One could reasonably argue that the rural Green Revolution has ultimately enabled the full realization of the urban industrial revolution. But how has this “agri-urban” transition been possible? This transition has been accomplished chiefly through innovation and technology, specifically the mechanization of agriculture, advances in crop germplasm, and breeding programs, as well as research and development of pesticides and fertilizers (Noone 1958; Alston et al. 2010; Popp et al. 2013). That is not to say there were no unintended consequences or examples of collateral damage in this historical transition. One needs to look no further than the dust-bowl (Baumhardt 2003) or Silent Spring (Carson 1962), but we have come a long way since then. The reality is that we need to produce food and we need to do so sustainably, which means producing more (food) from less (land).

With respect to agricultural pesticides (encompassing herbicides, insecticides, fungicides, and other chemicals designed to control agricultural pests), these compounds are biologically active by design, and thus their use comes with inherent risks, but these risks are now thoroughly evaluated and stringently regulated. Once established, the EPA phased-out the use of organochlorine insecticides (e.g., DDT) due to inadvertent adverse effects on the environment, particularly effects on birds (i.e., eggshell thinning). These compounds are persistent, bioaccumulative, and toxic (PBT), and as a result, subsequent generations of insecticide classes (including, chronologically, the organophosphates, pyrethroids, and neonicotinoids) have been developed in a continual effort to address these undesirable qualities and improve upon chemical safety. Under FIFRA, pesticides must undergo exhaustive scientific and regulatory review focused on potential ecological and human health effects before they can be registered for use, and are subject to ongoing and stringent review after registration. Despite these efforts, recent publications have attempted to implicate/associate neonicotinoids, either directly or indirectly, with declines in bird populations (Goulson 2018; Hallmann et al. 2014; Gibbons et al. 2015). Do these claims of cause and effect have merit or do they simply lack a more holistic historical context and perspective? This manuscript attempts to address this question by exploring the following considerations: (1) historical land use change within the USA, (2) comparing neonicotinoids with previous generations of insecticides, and (3) holistically evaluating causes/drivers of avian mortality. These considerations were explored by (1) mining historical land use data from the USDA Economic Research Service (ERS) in order to characterize relative land use change over the past ~ 70 years, (2) mining and comparing potency and fate data for four successive generations of insecticides (organochlorines, organophosphates, pyrethroids, and neonicotinoids), focusing specifically on avian and invertebrate toxicity, bioaccumulation/biomagnification potential, half-life, and rates, and (3) mining data sources which ranked causes/drivers of avian mortality.

## Historical and current land use

### Data sources and analysis

**Fig. 1:**
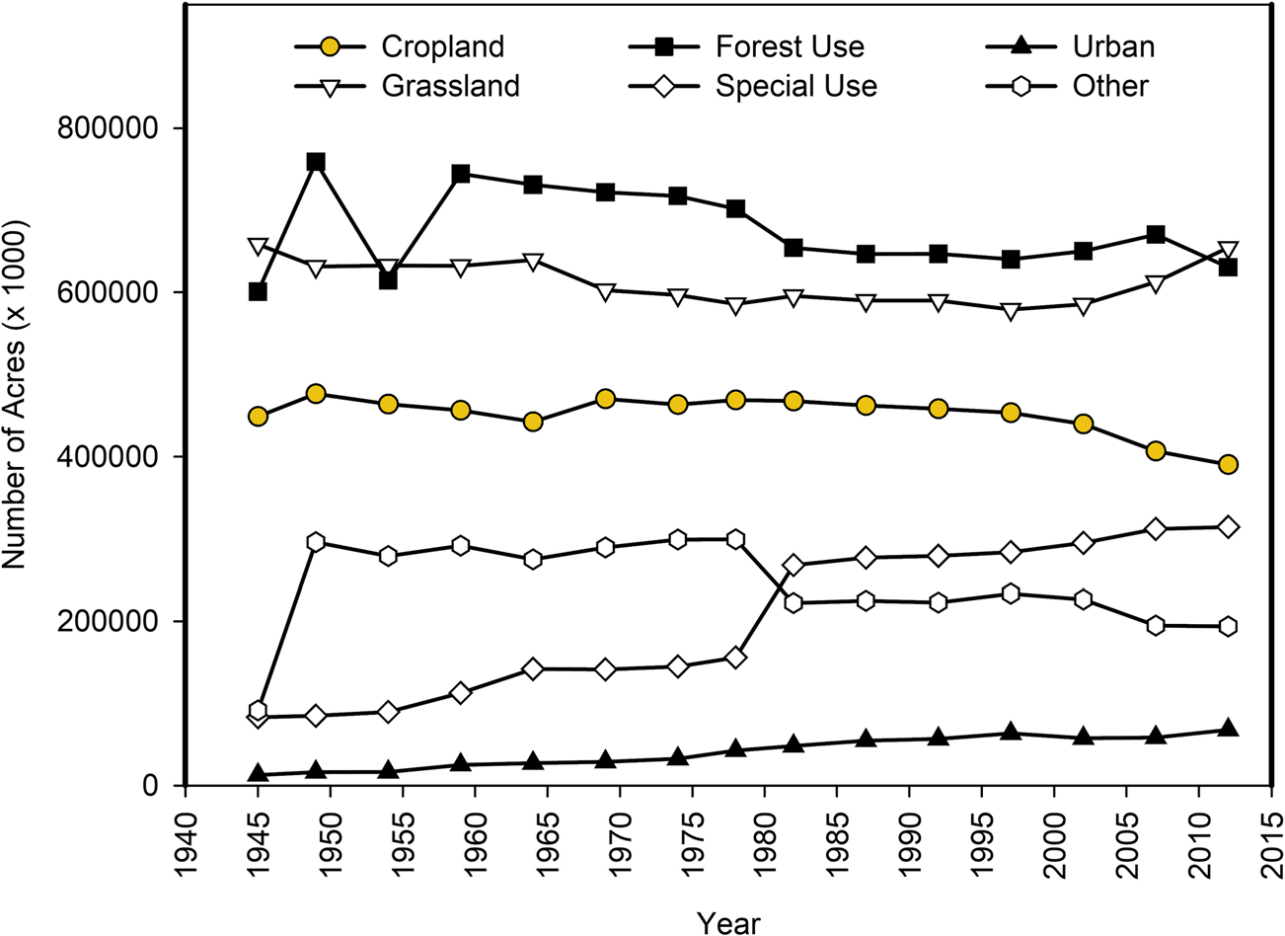
Trends in land use in the total USA from 1945 to 2012, as reported by USDA ERS (2018)

The first question we sought to address was whether land use patterns (particularly agricultural uses) changed significantly in the USA over the period documented by the USDA ERS (i.e., 1945 to present). For the purpose of these high-level land use pattern assessments, summary data from the USDA ERS (2018) were queried and grouped together by use type as defined by USDA (i.e., cropland, grassland pasture and range, forest use, urban, special uses, and other). The data set was organized by regional groupings (detailed in Osteen and Szmedra 1989). Trends were assessed by linear regression across the entire reporting period, with *p* < 0.05 deemed to be significant. Significant trends are summarized by region in Table 1, and data for the total USA are plotted in Fig. 1. Pesticide use data were primarily obtained from US EPA and USDA ERS sales and usage summary reports, particularly from Osteen and Szmedra 1989, Fernandez-Cornejo et al. (2014), Atwood and Paisley-Jones (2017), and Grube et al. (2011).

**Table 1:** Summary of land use changes in USDA ERS-defined regions between 1945 and 2012. Statistical significance of change over the time period was determined by linear regression, *p* < 0.05. *NS*, non-significant results

Region	Land use type	Rate of change (acres/year)	Direction	Region	Land use type	Rate of change (acres/year)	Direction
Total USA	Cropland	704,951	↓	Appalachian	Cropland	184,289	↓
	Grassland	560,678	↓NS		Grassland	42,361	↓NS
	Forest	757,151	↓NS		Forest	76,297	↑
	Special use	4,085,981	↑		Special use	48,275	↑
	Urban	858,504	↑		Urban	103,934	↑
	Other	194,504	↑NS		Other	20,636	↓NS
48 states	Cropland	705,575	↓	Southeast	Cropland	200,855	↓
	Grassland	565,066	↓NS		Grassland	1848	↓NS
	Forest	1,053,272	↓		Forest	33,675	↓NS
	Special use	1,262,110	↑		Special use	62,574	↑
	Urban	846,810	↑		Urban	147,567	↑
	Other	18,249	↑NS		Other	6914	↑NS
Northeast	Cropland	190,336	↓	Delta States	Cropland	43,754	↓NS
	Grassland	78,444	↓		Grassland	12,719	↓NS
	Forest	51,220	↑NS		Forest	26,085	↓NS
	Special use	64,892	↑		Special use	15,718	↑
	Urban	150,768	↑		Urban	34,279	↑
	Other	19,745	↓NS		Other	639	↑NS
Lake states	Cropland	93,605	↓	Southern plains	Cropland	64,018	↓NS
	Grassland	23,111	↓NS		Grassland	304,737	↑
	Forest	85,658	↓		Forest	432,443	↓
	Special use	58,807	↑		Special use	33,418	↑
	Urban	47,263	↑		Urban	87,991	↑
	Other	84,964	↑		Other	44,498	↑NS
Corn belt	Cropland	16,569	↓NS	Mountain	Cropland	96,109	↑
	Grassland	106,338	↓		Grassland	441,141	↓
	Forest	58,513	↑		Forest	245,400	↓
	Special use	17,751	↑		Special use	591,293	↑
	Urban	97,454	↑		Urban	69,120	↑
	Other	66,979	↓		Other	86,741	↓NS
Northern plains	Cropland	38,398	↑NS	Pacific	Cropland	46,658	↓
	Grassland	118,543	↓		Grassland	45,249	↓NS
	Forest	8296	↑NS		Forest	424,128	↓
	Special use	4211	↓NS		Special use	373,593	↑
	Urban	12,600	↑		Urban	95,850	↑
	Other	45,461	↑NS		Other	29,873	↑NS
Regions and their states (as per Osteen and Szmedra 1989)							
Northeast	Connecticut, Delaware, Maine, Maryland, Massachusetts, New Jersey, New Hampshire, New York, Pennsylvania, Rhode Island, Vermont						
Lake	Michigan, Minnesota, Wisconsin						
Corn Belt	Illinois, Indiana, Iowa, Missouri, Ohio						
Northern Plains	Kansas, Nebraska, North Dakota, South Dakota						
Appalachia	Kentucky, North Carolina, Tennessee, Virginia, West Virginia						
Southeast	Alabama, Georgia, Florida, South Carolina						
Delta	Arkansas, Louisiana, Mississippi						
Southern Plains	Oklahoma, Texas						
Mountain	Arizona, Colorado, Idaho, Montana, Nevada, New Mexico, Utah, Wyoming						
Pacific	California, Oregon, Washington

The authors of this paper acknowledge only one land use data set was used and that the categories used by USDA might be imperfect in some scenarios. As an additional caveat, because this case study focuses on the USA, analyses and conclusions may not necessarily be applicable to other regions, particularly developing countries. In part, this is due to potentially different socio-economic drivers for land use change, particularly agriculture, where ag-forest conversion may be more prevalent (Lambin and Meyfroidt 2011). There are also geospatial ecological linkages (e.g., migration) that do not recognize international borders and which may be difficult to account for. In addition, the data set provides the net change over time and does not allow the resolution to analyze parcel by parcel changes, so there may be more conversion than is captured in a single year. Nonetheless, the analysis was intended to provide a starting point for conversation around the many intersecting factors that might be driving changes in species abundance, particularly bird species, in the USA.

### Overall trends

The total land area in the USA is approximately 2.3 billion acres (Bigelow and Borchers 2012). In 2012, 29% of land was permanent grassland pasture and range, 28% was forest use, 17% was cropland, 14% was designated as special use (e.g., rural transportation, rural parks and wildlife, defense and industrial, and plus miscellaneous farm), 3% was urban, and the remaining 9% was designated as miscellaneous other. Alaska has relatively large areas of special use and other use, so when only the 48 contiguous states (excluding Alaska and Hawaii) are included, the proportions do shift to reflect this change (Bigelow and Borchers 2017; USDA ERS 2018).

In the 48 contiguous states, cropland and forest use areas decreased significantly over the period from 1945 to 2012, while special use and urban areas increased significantly (*p* < 0.05). Special use land designations have been the fastest growing land use type over the monitored period, and include rural transportation facilities, rural parks and wildlife areas (typically the largest portion of special use), defense and industrial areas, and farmsteads, roads, and miscellaneous farmland. In the total USA, a marked increase in special use areas in the 1980s (shown in Fig. 1) was largely the result of reclassification of non-commercial forested areas in Alaska (Frey and Hexem 1984). Forest use areas (which include forest for timber/forest products and forest for grazing) reached a peak around 1960 and have been in decline since, with the largest areas converted in the Southern Plains, Mountain, and Pacific regions (USDA ERS 2018).

### Agricultural trends

**Fig. 2:**
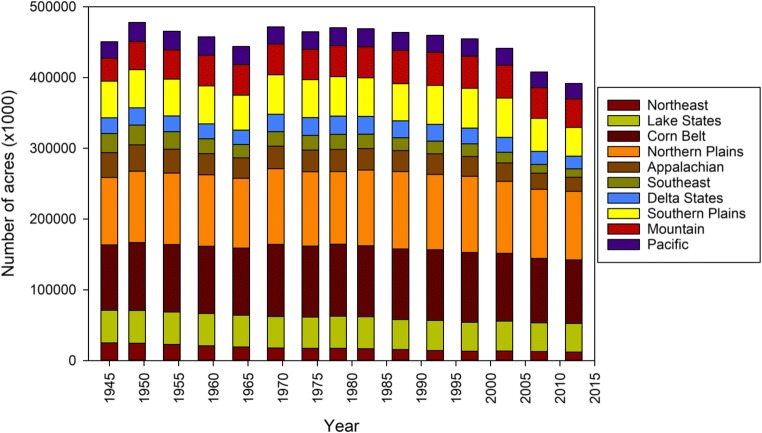
Trends in cropland area by region from 1945 to 2012 (Source: USDA ERS 2018)

The trends in cropland are plotted by region in Fig. 2. These demonstrate the contributions of each region to the total agricultural areas in the continental USA as a whole, as well as the trends within each region. It should be noted that the regions vary by relative size and number of states (as per Table 1), and that grassland comprises a larger portion of the total acreage in the USA than agriculture (Fig. 1) but also includes pasture and range areas (USDA ERS 2018). With regard to total cropland, the Mountain region is the only one where the amount of cropland significantly increased between 1945 and 2012. For all other regions, the total cropland area was either stable or decreasing over time. Grassland (and pasture) significantly decreased in four regions (Northeast, Corn Belt, Northern Plains, and Mountain), and remained stable in all others, with the exception of the Southern Plains, where a significant increase was reported (Table 1).

In addition to changes in the amount of land designated to cropland uses, there have also been shifts within the category and among different crops, which make the assessment of agricultural trends and environmental effects of agriculture much more complicated (Bigelow and Borchers 2012; Fernandez-Cornejo et al. 2014). Different crops face different pest pressures, have different requirements for irrigation and nutrient inputs, and have specific market demands and economic realities associated with them (Bigelow and Borchers 2012; Fernandez-Cornejo et al. 2014). As noted in the “Introduction” section, the reality of requiring agricultural production to keep up with changing consumer preferences and overall increasing demand due to population growth has resulted in intensified/consolidated agricultural practices on smaller total land areas (Noone 1958; Brown et al. 2005; Alston et al. 2010; Popp et al. 2013: Sutton et al. 2017), as is reflected in the data from USDA ERS (2018).

### Urban land use

In contrast to the declining trends seen in cropland use acreage, urban land use areas have steadily increased over time (USDA ERS 2018). From 1945 to 2012, urban land use grew at more than twice the rate of population increase over the same period (Bigelow and Borchers 2012). Although the urban acreage is by far the smallest land use category, demands for urban and (suburban) development will continue to increase, and these relatively “small” areas have outsized footprints. Brown et al. (2005) reported that exurban development (i.e., low-density urban development) expanded dramatically from 1950 to 2000, particularly in the Eastern Temperate Forest ecoregion. Likewise, Radeloff et al. (2005) examined the interface between wildlands and urban areas, noting that 9% of the land area and 39% of all houses in the conterminous USA are located within this interface. In Connecticut, up to 72% of land area could be described as having houses intermingling with wildland vegetation (Radeloff et al. 2005). This pattern of “rural sprawl” (as described by Brown et al. 2005), which is not necessarily housed within metropolitan counties, affects large parcels of land. The most attractive locations for these high-amenity developments are typically those with rich biodiversity since access to outdoor recreation and natural landscapes are main drivers for human consumers. Predictably, extensive development of such areas has unintended ecological consequences, such as habitat fragmentation, direct threats to wildlife, and biodiversity declines (Brown et al. 2005; Radeloff et al. 2005 and references therein). In addition to encroaching on sensitive ecoregions, urbanization often consumes the most productive agricultural land, which leads to resource challenges (Satterthwaite et al. 2010; Imhoff et al. 2004). For example, in terms of biologically available energy, the loss of net primary productivity from urban expansion over agricultural lands alone is estimated to be equivalent to the caloric requirement of 16.5 million people or ~ 6% of the US population (Imhoff et al. 2004). Thus, not only does urban sprawl exert production pressure on a shrinking agricultural landscape through land transformation but also through increased demand from population growth.

### Land use conclusions

In terms of the total amount of land strictly classified as cropland, there has been a steady or slightly declining trend across nearly all of the USA since 1945. Grassland or pastureland trends are more difficult to tease apart due to the nature of the data, but together, these areas have been stable over much of the continental USA, with significant decreases in four regions and an increase in one region. In contrast, urban areas have been steadily increasing, as well as the suburban or rural sprawl that accompanies urban development.

Any land use conversions from preferred habitat to an alternative will have impacts on the species residing there, both permanently and as part of a migratory route (Ewert and Hamas 1995). The more interesting (and challenging) question is how shifts in specific activities on the landscape within the same land use classification might have different effects on non-target species, including birds. Herein lies the question with regard to the effects of agricultural land use on biodiversity that is worthy of further exploration.

## Trends in pesticide use and potency

**Fig. 3:**
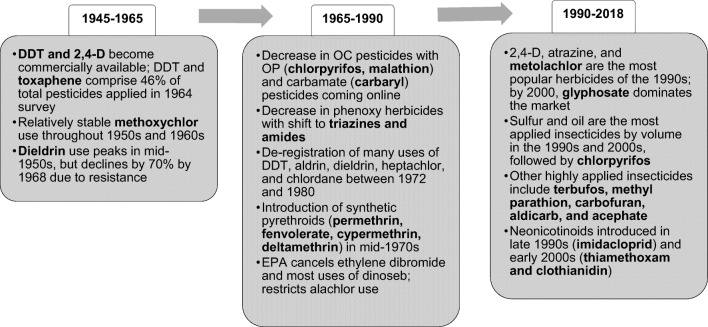
Chronology of agricultural pesticide development, use, and de-registration from 1945 to present. (Sources: US Department of Health, Education, and Welfare 1969; Osteen and Szmedra 1989; Atwood and Paisley-Jones 2017)

Because pesticides, specifically neonicotinoids, have been suggested as direct (e.g., overt oral/dietary toxicity) or indirect (e.g., cascading effects on food sources such as invertebrates) contributors to bird population declines by certain researchers (Goulson 2018; Hallmann et al. 2014; Gibbons et al. 2015), we next sought to explore trends in agricultural chemical use and properties over time. Key development and registration events and use trends are presented in Fig. 3 to demonstrate shifts in use patterns over time. In addition, a chronological comparison of major insecticide classes, based on avian toxicity (acute oral and chronic reproduction endpoints), environmental fate parameters, and application rates, is presented in Table 2.

**Table 2:** Chronological comparison of key insecticide classes based on avian toxicity (acute oral and chronic reproduction endpoints), environmental fate parameters, and application rates

Class/compound	Avian acute LD_50_ (mg/kg bw)^1^	Avian chronic NOEC (mg/kg diet)^1^	Honeybee acute contact LD_50_ (μg a.i./bee)^2^	Log K_ow_	Aerobic soil half-life (days)	Max single application rate for Ag (lb a.i./A)^8^	Source(s)
Organochlorines (1940s to 1972)							
DDT^3^	841	0.3	7.12	7.48	730 to 5475	1.2 to 12	WHO (1989); Lincer (1975); FAO (1967); Atkins et al. (1975)
Aldrin^4^	6.59	< 0.5	0.353	6.5	~ 53	0.5 to 5	EPA (1986); DeWitt (1956); ASTDR (2002); Martin (1968); Atkins et al. (1975)
Dieldrin^5^	6.9	< 0.5	0.139	6.2	~ 913	0.1 to 1.5	McEwen and Brown (1966); DeWitt (1956); ATSDR (2002); Brooks (1974); Andersen and Weihing (1959); Atkins et al. (1975)
Heptachlor^6^	2080	< 0.1	0.562	5.44	730 and 5110	2.5 to 10	Hudson et al. (1984); Wagstaff et al. (1981); ATSDR (1989); EPA (1960); Atkins et al. (1975)
Organophosphates (1960s to present)							
Chlorpyrifos^7^	5.62	25	0.059	4.7	19 to 297	0.3 to 6	EPA (2018a)
Malathion^7^	136	110	0.156	2.8	0.3 to 7	0.156 to 7.5	EPA (2018b)
Diazinon	1.44	8.3	0.41	3.77	9 to 57	0.25 to 5	EPA (2018c)
Methyl Parathion	6.6	6.3	0.111	2.86	11.25	0.5 to 2	EPA (2009); ATSDR (2001)
Pyrethroids (1970s to Present)							
Bifenthrin	1800	75	0.015	6.4	97 to 250	0.04 to 0.5	EPA (2010a)
Permethrin	> 2000	125	0.024	6.1	8.70 to 305	0.007 to 0.4	EPA (2011); EPA (2016a)
Lambda-Cyhalothrin	> 3950	5	0.038	7	28.2 to 60.5	0.015 to 0.156	EPA (2010b)
Cypermethrin	> 2000	50	0.023	6.4	12.7 to 170	0.025 to 0.1	EPA (2012); EPA (2016b)
Neonicotinoids (1990s to Present)							
Thiamethoxam	576	300	0.024	− 0.13	34.3 to 464	0.17 to 0.27	EPA (2017a); EPA (2017e)
Imidacloprid	33	125	0.043	0.57	172 to 608	0.043 to 0.25	EPA (2017b); EPA (2016c)
Clothianidin	423	205	0.0275	1.12	144 to 5357	0.05 to 0.2	EPA (2017c); EPA (2017e)
Dinotefuran	> 2250	2150	0.047	− 0.549	9 to 113	0.068 to 0.54	EPA (2017d); EPA (2017f)

^1^Either Northern bobwhite quail (*Colinus virginianus*), Japanese Quail (*Coturnix japonica*), or Mallard duck (*Anas platyrhynchos*) unless otherwise indicated

^2^European honey bee (*Apis mellifera*) unless otherwise indicated

^3^Note the chronic endpoint for DDT is derived from the metabolite DDE and is defined by statistically significant (15%) eggshell thinning in the American kestrel (*Falco sparverius*) at 3 mg/kg diet but not 0.3 mg/kg diet; see Lincer (1972) in WHO (1989)

^4^Note that Aldrin converts to Dieldrin in soil systems (ASTDR 2002)

^5^Acute endpoint for the sharp-tailed grouse (*Tympanuchus phasianellus*)

^6^Half-life values for heptachlor and heptachlor-epoxide, respectively; chronic avian endpoint for broiler chickens (*Gallus gallus domesticus*); use rates obtained from EPA archive file (heptachlor label) dated 1960 (precedes EPA establishment in 1970)

^7^Acute endpoint for Ring-Necked Pheasant (*Phasianus colchicus*)

^8^For metric equivalents in kg a.i./ha multiply by 1.12

Generally, the organochlorines (e.g., 2,4-D, DDT, methoxychlor, dieldrin) dominated the market from 1945 to the early 1970s. The de-registration of uses of DDT led to the adoption of new insecticide chemistries such as organophosphates (OPs) (e.g., chlorpyrifos and malathion) and carbamates (e.g., carbaryl) (U.S. Department of Health, Education, and Welfare 1969; Osteen and Szmedra 1989). Pyrethroid insecticides (e.g., permethrin) were introduced in the 1970s and gained market shares by the early 1980s, followed by the introduction of neonicotinoids in the late 1990s and early 2000s, with rapid adoption of those chemistries by the agricultural sector (Osteen and Szmedra 1989; Grube et al. 2011; Atwood and Paisley-Jones 2017). By 2008, imidacloprid became the most used insecticide globally (Simon-Delso et al. 2015). The popularity of OPs has also persisted to the present, and among pesticides overall, the herbicide glyphosate steadily became the most applied compound by 2000 (Grube et al. 2011; Atwood and Paisley-Jones 2017; Simon-Delso et al. 2015).

In 2008, corn received 39% of the volume of pesticides applied to crops in the USA; soybeans, potatoes, and cotton received 22%, 10%, and 7% of active ingredients applied that year, respectively (Fernandez-Cornejo et al. 2014). Taken together, these four crops accounted for 78% of pesticide use and can be used as representatives of crops and pesticide applications as a whole. As reported by Fernandez-Cornejo et al. (2014), the chemical potency of pesticides towards their targets has generally increased from the 1970s to the 2000s, as demonstrated by reduced application rates. Chronic toxicity to non-target species has declined, largely as a result of policy changes to ban compounds such as DDT, toxaphene, and aldrin in the 1970s and 1980s. With these bans, there was also a reduction in persistence (i.e., half-life in soil), followed by an increase in the 1990s with the growing use of metolachlor and pendimethalin, among others. Since the early 2000s, persistence has steadily declined, first as a result of increased glyphosate use and then with the introduction of neonicotinoid insecticides (Fernandez-Cornejo et al. 2014). These same trends can be seen in Table 2 where acute and chronic toxicity and environmental persistence characteristics are compared among classes and compounds. The exception is the honey bee acute contact LD_50_, which is lower for pyrethroids and neonicotinoids than OCs and OPs as a result of the mode of action and target organisms for these insecticidal compounds. Potential risks posed by acute contact toxicity are, by nature, generally easier to assess, regulate, and control.

**Fig. 4:**
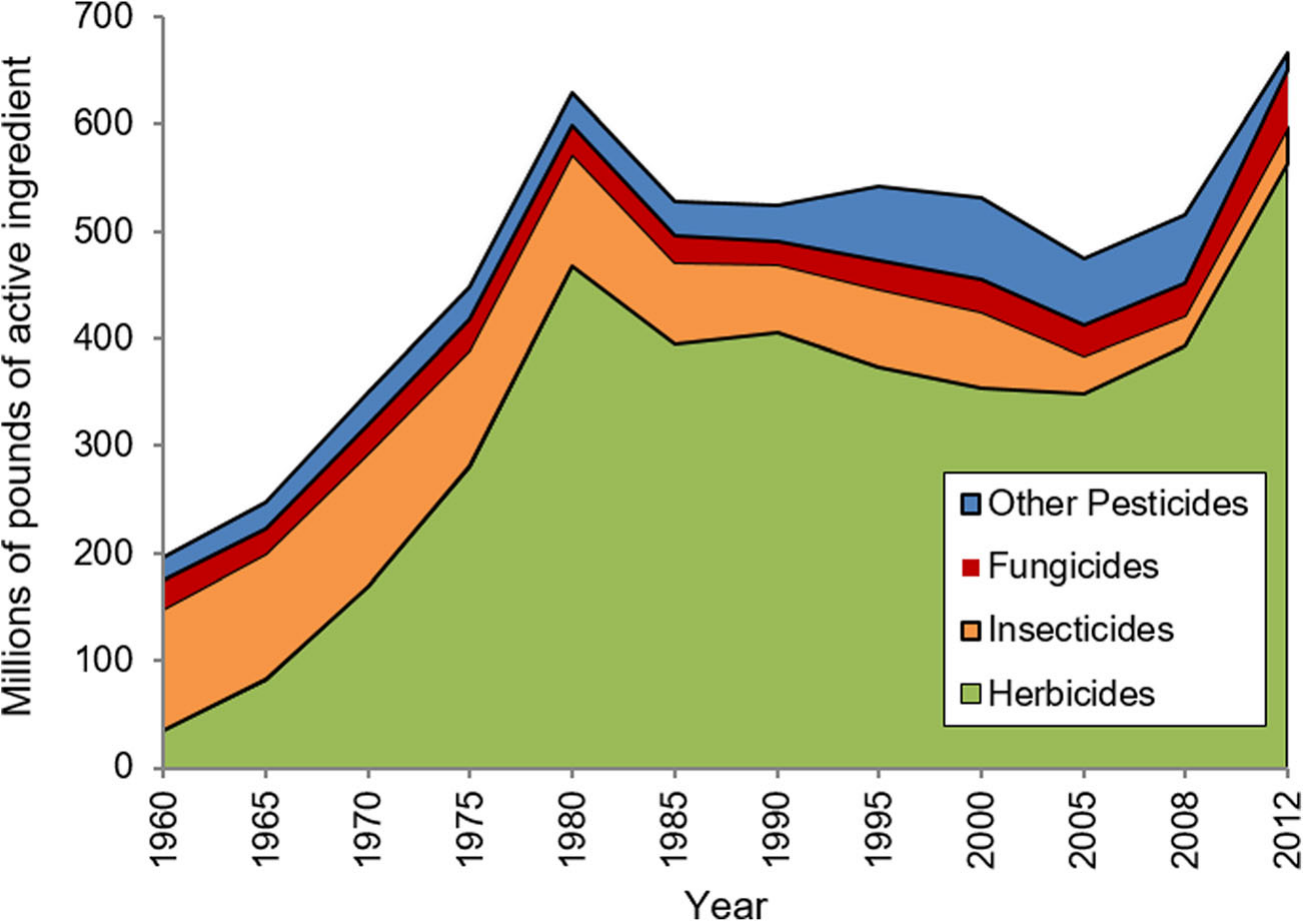
Pesticide use (herbicides, insecticides, fungicides, and others (i.e., sulfur and oils, fumigants, and other pesticides)) in US Agriculture from 1960 to 2012. (Sources: Fernandez-Cornejo et al. 2014; Atwood and Paisley-Jones 2017)

As seen in Fig. 4, herbicides comprise the bulk of active ingredients currently applied to agricultural fields in the USA, and this has been true since the 1970s. The total amount of herbicides increased from 2005 to 2012, during which time applied volumes of insecticides and other pesticide active ingredients decreased, and fungicide use remained relatively stable (Fernandez-Cornejo et al. 2014; Atwood and Paisley-Jones 2017). In the 1950s and 1960s, insecticides comprised the majority of active ingredients applied, but volumes have decreased dramatically over time (Fig. 4). This is largely a result of new chemistries (particularly the neonicotinoids) with greater efficacy towards target pests (Table 2).

Overall, regulation and oversight for pesticides have strengthened over time, resulting in trends towards less persistent, more efficacious agricultural chemicals. Herbicide applications have grown to comprise a greater proportion of the chemicals applied to agricultural fields (based on bulk active ingredient), and insecticides are as targeted as they have ever been. With the inherent toxicity of pyrethroids and neonicotinoids towards some non-target insects, including bees, there is cause for conservative and responsible use of these compounds, but on the whole, trends in pesticide chemistries seem to be moving in a positive direction. Increasing use patterns over a relatively stable or even decreasing cropped area does suggest agricultural intensification that should be closely monitored. This should also be part of a broader conversation around consumer demands for specific products with greater pest pressures and integrated pest management approaches in the future reality of producing more food with fewer resources.

## Case study: avian species in the USA

**Fig. 5:**
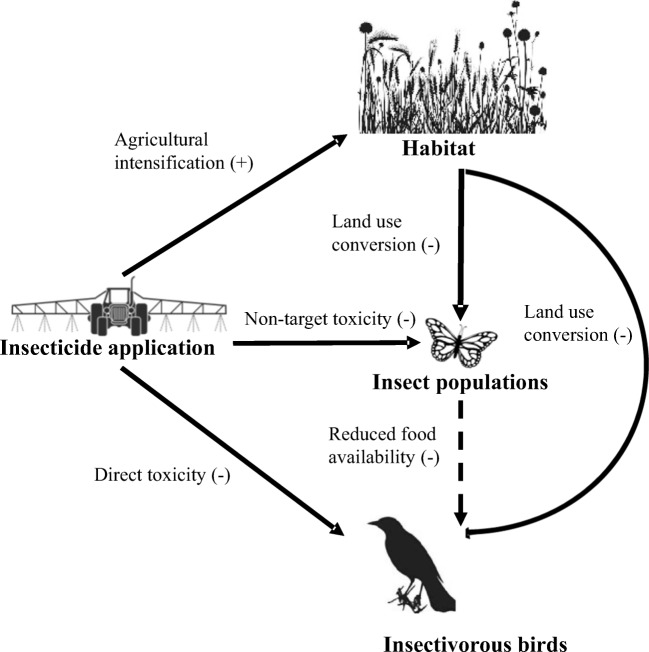
Conceptual figure of mechanisms by which neonicotinoids and habitat status are proposed to directly (solid line) or indirectly (dashed line) impact birds

For the purpose of this analysis, rather than attempt to tackle overall species diversity changes in response to the interplay of land use shifts and changing agricultural pressures, we chose to focus on avian species as our case study. This also follows from the arguments raised by Goulson (2018) and others with particular regard to neonicotinoids and their potential direct and indirect effects on birds (Fig. 5). We first sought to understand what the prevailing trends in bird species in the USA were over the timeline coinciding with agricultural intensification and urbanization. We were interested in whether there were regional trends or life history traits that were common among species experiencing severe declines and could perhaps be predictive of population trends. In particular, we sought to explore whether reported avian declines corresponded to land use trends, especially those related to agriculture and the associated trends in pesticides. As for the land use assessment in the “Historical and current land use” section, we acknowledge that this literature review and assessment is not exhaustive, but rather is intended it to be a starting point for further conversations.

### Avian trends, 1966–2015

Data used to assess trends over time were collected by the United States Geological Survey (USGS) Breeding Bird Survey (BBS) between 1966 and 2015. Analyses were performed by Sauer et al. (2017), and results of those analyses are summarized here without further data evaluation. In the USA, 36% of surveyed bird species (*n* = 419 total) experienced significant populations declines between 1966 and 2015. For comparison, 25% of species experienced significant population increases over the same time period (Sauer et al. 2017). When data were limited to the period from 2005 to 2015 and re-analyzed, 13% of species had significant declines and 22% increased significantly (Sauer et al. 2017), as summarized in Table 3 based on habitat, nesting type and location, and migration pattern. The overall observed trend of slightly more decreasing species than increasing species is consistent with other observations of regularly occurring native bird species in Canada (NABCI 2012) and the USA (Belden et al. 2018).

**Table 3:** Population changes in birds grouped by habitat, nesting practices, and migration pattern for species demonstrating statistically significant population trends by analysis of BBS data (*p* < 0.05) (as reported by Sauer et al. 2017). Number and percent of total species within each grouping that were significantly increasing or decreasing over the periods from 1966 to 2015 and 2005 to 2015 are reported

Group/guild	Total species	1966–2015				2005–2015			
		Declining		Increasing		Declining		Increasing	
		**%**	**#**	**%**	**#**	**%**	**#**	**%**	**#**
Grassland	28	54	15	4	1	18	5	4	1
Wetland	86	22	19	27	23	3	3	27	23
Woodland	132	31	41	33	44	11	15	26	34
Successional scrub	87	48	42	15	13	20	17	14	12
Urban	15	60	9	27	4	40	6	20	3
Cavity nesting	62	29	18	35	22	5	3	31	19
Open-cup nesting	182	45	82	20	36	19	35	16	29
Short distance migrant	107	41	44	24	26	21	22	20	21
Permanent resident	93	27	25	31	29	11	10	19	18
Neotropical migrant	136	45	61	22	30	15	20	24	33
Ground or low nesting	113	50	57	15	17	23	26	13	15
Mid-story or canopy nesting	123	36	44	28	34	15	18	22	27

Different species are experiencing different stressors and outcomes (Berg et al. 2015; Stanton et al. 2018; Sauer et al. 2017; Belden et al. 2018) so that reporting overall trends in bird populations can obscure some of the underlying trends. According to the BBS data and analysis by Sauer et al. (2017), there are differences among different habitats whereby species that predominately frequent grassland, successional scrub, and urban habitats are more likely to be declining than increasing over the period from 1966 to 2015. Likewise, North American Bird Conservation Initiative (NABCI) (2012); (NABCI) (2016) found that waterfowl and forest birds in Canada were generally stable or slightly increasing in their overall populations, while grassland species were in steeper decline overall. In contrast, for the same period, US wetland and woodland species had nearly equal numbers of species experiencing population increases as declines (Sauer et al. 2017). From 2005 to 2015, wetland and woodland species were actually more likely to be increasing than decreasing (Sauer et al. 2017). Belden et al. (2018) similarly reported nearly equal numbers of species increasing as decreasing in high-intensity cropland regions based on BBS data from 1995 to 2016.

When nesting types were considered, open-cup nesters were more often in decline over the longer time period but had more stable populations over the shorter term (Sauer et al. 2017). Cavity nesters had approximately 30% of species experiencing significant increases or declines in population from 1966 to 2015, while the remaining 40% of species remained stable. In the period from 2005 to 2015, nearly all species were either increasing or remaining stable, with only 5% in decline. Ground- or low-nesting species were more likely to be in decline than increasing for both the longer and shorter periods, but the majority of species were relatively stable from 2005 to 2015. Mid-story or canopy nesters had fairly equal numbers of increasing and decreasing species over both periods (Sauer et al. 2017).

Migration is an energetically expensive endeavor with many opportunities to encounter human and natural stressors (Newton and Brockie 2008 and references therein), and more species of short distance and neotropical migrants experienced declines in both the short and long term than permanent residents (Sauer et al. 2017). The NABCI also found that species overwintering in the USA fared better than those traveling to Mexico, the Caribbean, and South America (NABCI 2012). However, most studies in North America have suggested that declines are more likely to be a result of changes to breeding habitats, as opposed to wintering habitats (Newton and Brockie 2008 and references therein).

Diet was not assessed by Sauer et al. (2017), but other studies suggest that differences exist among species with different dietary preferences. For example, an analysis of grassland- and farmland-dependent bird population data from BBS and elsewhere was conducted by Stanton et al. (2018) to identify drivers of declines in these populations. The authors found that farmland birds were in steeper decline than birds associated with all other biomes, with 74% of species decreasing between 1966 and 2013. Stanton et al. (2018) concluded that the aerial insectivore guild was experiencing the most significant declines, which were attributed to indirectly to reduced flying insect prey populations. In contrast, an analysis of BBS data from 1995 to 2016 in the context of local land cover change reported relatively consistent declines across feeding guilds, and in both cropland and grassland areas (Belden et al. 2018). Although most bird species were found to exhibit significant responses to land cover changes, the proportion of species demonstrating positive or negative responses was comparable in all cases and did not appear to fall into any particular foraging guild (Belden et al. 2018).

The ten species for which analyses of the 1966 to 2015 trend data suggested the most significant declines are summarized in Table S1, including information about diet, habitat, migration, and threats from the Cornell Lab of Ornithology’s *Birds of North America* (Rodewald 2015). These species represent a range of different habitats, nesting strategies, migratory behaviors, and species-specific susceptibilities to natural and anthropogenic stressors and threats (Rodewald 2015). While closer examination of these species might not provide a “smoking gun,” it does demonstrate a few key points: (1) The importance of monitoring programs and appropriate incorporation of citizen science into long-term monitoring data sets (especially for rare/elusive species) (Sauer et al. 1996); (2) Birds with very specific habitat/dietary/breeding requirements will likely face greater challenges than generalists (Chiron et al. 2014; Stanton et al. 2018); and (3) Cumulative effects assessment is crucial for fully characterizing mortality risks and developing conservation strategies for bird species (Newton and Brockie 2008).

### Threats to birds

No matter the current land use in question, human or naturally induced changes will have some effect on the species inhabiting those areas, and often there will be multiple simultaneous stressors. Sauer et al. (1996) cautioned that simple explanations for changes in bird populations as reported by the BBS are bound to be controversial due to regional variations in populations and environmental conditions. As demonstrated by the information in Table S1, there are many different factors that threaten survival of birds. These include many human-driven activities, such as shifts in land use, urban development, etc., as well as natural competitive behaviors, weather events, and predators (Sauer et al. 1996; Rodewald 2015).

#### Human-caused threats

In Table 4, the top human-caused threats to birds in the USA are summarized according to data from the U.S. Fish and Wildlife Service (2017). These estimates are not necessarily definitive or meant to dismiss other indirect causes (since these are often challenging to accurately document); rather, they are intended to provide context for a broader conversation. Based on the median estimates from Loss et al. (2014a, 2014b), domestic cats are by far the greatest human-related threat to birds, followed by collisions with building glass, and collisions with vehicles. Pesticides (collectively) accounted for a relatively small portion (2.2%) of recordable bird deaths based on this data set (U.S. Fish and Wildlife Service 2017; Pimentel 2005). Moreover, “pesticides” are not further broken out to differentiate agricultural chemicals from other types of pesticide use (e.g., residential, golf courses, industrial or commercial properties).

**Table 4:** Estimates of mortality for the top human-caused threats to birds in the USA (Source: US Fish and Wildlife Service 2017)

Hazard/type	Min. range	Max. range	Median/avg. estimated	Percent of total	Reference
Cats	1,400,000,000	3,700,000,000	2,400,000,000	72.2	Loss et al. 2014a
Collision—building glass	365,000,000	988,000,000	599,000,000	18.0	Loss et al. 2014a
Collision—vehicle	89,000,000	340,000,000	214,500,000	6.5	Loss et al. 2014b
Poison	n/a	n/a	72,000,000	2.2	Pimentel 2005
Collision—electrical lines	8,000,000	57,300,000	25,500,000	0.8	Loss et al. 2014c
Collision—communication towers	n/a	n/a	6,600,000	0.2	Longcore et al. 2012
Electrocutions	900,000	11,600,000	5,600,000	0.2	Loss et al. 2014c
Oil pits	500,000	1,000,000	750,000	0.02	Trail 2006
Collisions—land-based wind turbines	140,438	327,586	234,012	< 0.01	Loss et al. 2014b
Habitat loss/conversion	n/a	n/a	n/a		
Collisions—offshore wind turbines	n/a	n/a	n/a		
Collisions—solar panels	n/a	n/a	n/a		
Burning—solar towers	n/a	n/a	n/a		
All	1,863,540,438	5,098,227,586	3,324,184,012	100.0	
All (excluding cats)	463,540,438	1,398,227,586	924,184,012	27.8	
Industry only (excluding cats and vehicles)	374,540,438	1,058,227,586	709,684,012	21.3	

Household and feral cats are representative of interactions between a natural stressor (i.e., predation) and human behaviors (i.e., high density of urban cats as pets, laissez-faire or Trap-Neuter-Return approach to feral colonies, and allowing free roaming by both owned and un-owned cats), and this interaction represents perhaps the largest human-related direct cause of mortality in birds (Blancher 2013; Bonnington et al. 2013; Loss et al. 2013a, b; Loss et al. 2014a). In addition to feline predators, there is evidence from a UK study that other predator species (e.g., Red Fox, *Vulpes vulpes*) may also be responsible for limiting populations of some bird species, particularly seabirds, gamebirds, and waders (Roos et al. 2018).

A review of trends in farmland birds and drivers of decline within an agricultural context found that 41.8% of studies reported negative effects from pesticides, 33% from habitat fragmentation or loss, 13.9% from harvesting operations or mowing, 9% from grazing disturbance, and 3.3% from reduced food availability (Stanton et al. 2018). These results suggest that both pesticide use and habitat loss are the most likely potential drivers of declines in farmland species (Stanton et al. 2018), which was also observed by Chiron et al. (2014). In contrast, Belden et al. (2018) found that nearly all of 31 studied species responded significantly to localized (i.e., within 2 km) land use changes, but most species had consistent, or increasing, abundance with agricultural intensification. This weighs against the potential association of agricultural pesticide with declines in bird populations. Berg et al. (2015) investigated whether land use changes in Sweden resulted in changes in farmland bird populations in an agricultural setting. The authors found that both land use changes and landscape characteristics (e.g., forest cover and heterogeneity) affected bird populations, but effects were very species-specific (Berg et al. 2015).

#### Direct incidents

We also queried wildlife incident data from the Ecological Incident Information System and summarized the results from 1981 to 2014 in Table 5 (EPA 2017g). Incidents, by nature, are relatively rare events with varying degrees of severity (i.e., number of mortalities). Also, these data only include incidents that were reported. Granted, the reporting system is not likely comprehensive, and conclusions based upon these data should be made cautiously, but they do provide a basis for comparison across chemical classes nonetheless. In total, there were 275 incidents and 102,046 reported wildlife mortalities (> 99% were avian species) from 1981 to 2014 (EPA 2017g). By number of mortalities, the top three chemical causes of bird incident mortality were sodium hydroxide, carbamates, and Avitrol (which is actually intended to cause acute toxicity in birds). In the case of sodium hydroxide, there was only one large (40,000 birds) poisoning event in the period from 1981 to 2014, so ranking this compound as most problematic is not necessarily representative. Likewise, carbamate and Avitrol each had one large poisoning event (≥ 20,000 birds each) comprising significant portions of the total mortalities documented. After Avitrol, the next most common cause of avian incidental poisoning was exposure to organophosphate compounds (as a single group). There were 171 incidents and 9123 bird mortalities where OPs were the confirmed or suspected causes, representing 65% of the total reported incidents and 9% of total mortalities.

**Table 5:** Chemical-related avian incidents and mortalities in the USA, as reported in the Ecological Incident Information System (EIIS) from 1981 to 2014

		Mortalities by year							
Principal cause of mortality	Total incidents	1981–1985	1986–1990	1991–1995	1996–2000	2001–2005	2006–2010	2011–2014	Total
Sodium hydroxide	1	0	0	0	0	40,000	0	0	40,000
Carbamate*	26	152	506	491	27,316	36	88*	375*	28,964
Avitrol	6	0	0	0	20,008	4	26	20	20,058
Organophosphate	171	1277	1771	2971	930	1751	151	272	9123
Chlorpyrifos	9	0	0	952	11	10	0	15	988
Strychnine	15	10	11	164	135	0	210	207	737
Baygon	1	0	500	0	0	0	0	0	500
Cyanide	6	0	36	25	0	200	38	0	299
Rodenticide	5	0	100	0	1	60	77	0	238
Baytex	1	225	0	0	0	0	0	0	225
Starlicide	2	0	0	93	100	0	0	0	193
Urea	1	0	0	0	0	170	0	0	170
Zinc phosphide*	9	0	0	10	0	14	76*	14	114
CHE-inhibiting compound	6	0	0	0	6	96	6	0	108
Thimet*	1	0	70*	0	0	0	0	0	70
Boron	1	0	0	0	0	0	39	0	39
Chlorinated pesticide	1	0	11	0	16	4	0	0	31
Nemacur	1	0	30	0	0	0	0	0	30
Brodifacoum	2	0	0	5	25	0	0	0	30
Pesticide	1	0	0	0	0	0	0	25	25
Carbon monoxide	1	0	0	0	0	0	0	25	25
Avicide	1	0	0	0	0	0	0	21	21
Nitrate	1	0	0	0	0	20	0	0	20
Chlordane*	3	0	0	0	13*	0	0	0	13
Hydrogen sulfide	1	0	10	0	0	0	0	0	10
Bromadiolone	1	0	0	0	8	0	0	0	8
DDT	1	0	0	0	7	0	0	0	7
Annual mortality total	275 incidents	1664	3045	4711	48,576	42,365	711	974	102,046

Reported mortality may have been linked to multiple causes; in some cases, cause of death could not be confirmed but mortality count is included under the suspected cause; reported mortalities also occasionally include mammalian species (maximum of 104 total, indicated by *)

Between 1996 and 2005, a handful of events resulted in over 90,000 mortalities, while from 2006 to 2014, there were only 1700 mortalities. These patterns and potential data gaps make it difficult to assess trends over time, and to determine whether specific compounds warrant special action or re-evaluation. Notwithstanding, subsequent to the widespread adoption of the neonicotinoid class of insecticides in the early to mid-2000s, the number of reported bird mortalities suspected to be induced by insecticides dropped dramatically (Table 5; EPA 2017g). Keeping in mind the potential limitations of the incident reporting system, this provides some evidence that neonicotinoids are not causing significant direct bird mortalities, and that even with widespread pesticide use, major poisoning events are rare.

Nevertheless, Millot et al. (2017) reviewed wildlife mortality incidents reported between 1995 and 2014 by the French SAGIR Network (national network for the surveillance of the health status of wildlife) where residues of imidacloprid were suspected or detected upon toxicological analysis. Bird species, particularly gray partridges (*Perdix perdix*) and “pigeons” (*Columba palumbus*, *Columba livia*, and *Columba oenas*) were among the most common species involved in these incidents, and the authors deemed that imidacloprid-treated seeds were at least a “likely” cause of mortality in 70% of the incidents. Millot et al. (2017) suggests there is evidence that birds are consuming pesticide-treated seeds and that mitigation measures might not be fully effective, but unfortunately, the subset of data used for the analysis is from a database of unknown size, so it is difficult to put these numbers in context with other incident causes for a more complete view of risks to birds. Overall, the number of incidents (101, totaling 734 dead animals) reported by Millot et al. (2017) for France in over 20 years suggests that lethal toxicity due to consumption of imidacloprid-treated seed is a relatively rare occurrence.

Moreover, toxicity and dosage assessments indicate that sufficient consumption of treated seeds to induce a toxic effect is, depending on the species, unlikely or impossible. While it is theoretically conceivable that a medium-sized bird (EPA characterizes birds into small, medium, and large categories of 20, 100, and 1000 g, respectively) could consume a lethal dose of imidacloprid from exposed seed, the risk is *de minimis* for other sizes of birds and other neonicotinoids. The number of seeds required for a bird to consume in order to exceed the corresponding toxicity threshold (i.e., LD50), referred to as the seed of concern, can be calculated based on the EPA memorandum regarding refinements for risk assessment of pesticide-treated seeds (EPA 2016d). Using a maximum seed application rate (mg a.i./kg seed) of 4220 for imidacloprid on corn (EPA 2017b), a dose of ~ 1.14 mg a.i./corn kernel (assuming a corn kernel mass of 270 mg) can be calculated. Using the acute oral LD_50_ for the Japanese quail (33 mg a.i/kg-bw; EPA 2017b) and the average mass of a Rock Pigeon (*Columba livia*) (355 g; Lowther and Johnston 2014) and a Gray Partridge (*Perdix perdix*) (397 g; Carroll 1993), ≥ 10 to 11 treated corn seeds would need to be consumed in order to potentially receive a dose exceeding the LD_50_. Conservatively, for clothianidin and thiamethoxam, which have substantially higher acute avian oral LD_50_ endpoints of 423 (EPA 2017c) and 572 (EPA 2017a), respectively, the corresponding number of seeds would be ≥ 120 and ≥ 160, which would be physically implausible for a pigeon or partridge to consume on an acute basis.

#### Indirect effects

Insecticides are specifically designed to control insect pests in a variety of situations, but predominantly in support of boosting agricultural yields and protecting human health. These compounds can also pose potential risks to non-target invertebrates, albeit thoroughly characterized and evaluated risks (USEPA 2014). The prospect of these potential risks raises the question of whether bird population status may be influenced indirectly by potential insecticidal effects on prey food resources (specifically insectivorous birds). Given the intended targets (insects), there is a plausible hypothetical scenario whereby insecticide use could reduce insect biomass in an agro-ecosystem and indirectly affect insectivorous bird populations through reduced food resources. Several studies have suggested such a mechanism to explain bird declines (Hallmann et al. 2017; Shortall et al. 2009; Ewald et al. 2015; Gibbons et al. 2015); however, other factors have also been implicated including light pollution (Grubisic et al. 2018; Hölker et al. 2010), climate change (Ewald et al. 2015; Frampton et al. 2000; Kingsolver et al. 2011; Sharma and Prabhakar 2014), urbanization (Jones and Leather 2012; Dennis et al. 2017), and vehicle traffic (Baxter-Gilbert et al. 2015). Thus, it is unclear if and to what extent insecticide use in agriculture has or is indirectly contributing to changes in cropland bird population status as a function of food resource. Potential causes of insect population declines are considered below for context.

Hallmann et al. (2017) re-analyzed data from a study (Sorg et al. 2013) evaluating insect biomass captured using Malaise traps placed in 63 German nature reserves. They concluded there was a seasonal decline of 76% and mid-summer decline of 82% in flying insect biomass over the 27 years. Agricultural intensification was associated by the authors with the overall decline of biodiversity in plants, insects, birds, and other species over the monitoring period (Hallmann et al. 2017). However, as indicated by Leather (2018), Hallmann et al. (2017) did not causally link the observed declines with pesticide use. Shortall et al. (2009) evaluated changes in total aerial insect biomass in the UK over 30 years (1973 to 2002), identifying a significant decline at one site (Hereford) but not at three other sites (Rothamsted, Starcross, and Wye). The authors cited increasing evidence of indirect effects of insecticides on birds but acknowledged there was no consistency identified temporally across sites regarding historical land use for agriculture. Based on 42 years of invertebrate monitoring data collected from cereal fields in the UK, Ewald et al. (2015) suggested that pesticide use was a more important explanatory variable than climate change, yet, of the 26 invertebrate taxa evaluated, fewer than half showed a decrease in abundance, while the other orders showed no consistent pattern or increased over the study (Leather 2018).

Ecological light pollution from artificial light alters natural light regimes in both aquatic and terrestrial ecosystems by disrupting interspecific interactions that have evolved in natural patterns of light and dark, resulting in serious consequences for community ecology (Langcore and Rich 2004; Garrett et al. 2019). Moths, beetles, true-bugs, lacewings, crane flies, midges, caddisflies, hoverflies, wasps, and bush crickets are all insect groups attracted to light (Langcore and Rich 2004 and references cited therein). In a review by Grubisic et al. (2018), the authors concluded that light pollution causes numerous disruptive and fitness-relevant impacts on both diurnal and nocturnal insects. This is especially important in agricultural landscapes due to ecosystem services rendered and other environmental pressures experienced by these organisms. As indicated by Hölker et al. (2010), many insects actively congregate around light sources until they die of exhaustion. Consequently, light pollution can adversely affect insect biomass and population size and modify the relative composition of insect populations, which can cause cascading effects further up the food chain (Hölker et al. 2010). As reported by Garrett et al. (2019), light pollution already occurs in over two-thirds of Key Biodiversity Areas globally, and generally higher human population density and GDP are associated with changes in night-time light conditions. With predicted increases in human population over the next century, the impacts of light pollution on aquatic and terrestrial organisms are expected to increase as well (Garrett et al. 2019).

As described by Kingsolver et al. (2011, and references cited therein), climate change varies with latitude, continent, season, and the diel cycle, where extreme weather events vary spatially and temporally rather than heterogeneously. Moreover, conditions approaching upper thermal limits for insects may affect juvenile survival and adult reproduction differently, where high temperatures preventing survival to maturity can reduce adult reproduction in both temperate and tropical regions (Kingsolver et al. 2011). This suggests that considering differential responses of multiple life stages is crucial to understanding the ecological and evolutionary consequences of climate change (Kingsolver et al. 2011). Moreover, margins of thermal safety limits for insects may be smaller than previously indicated, potentially further decreasing the fitness and persistence of insect populations in both tropical and temperate regions (Kingsolver et al. 2011). Consequently, extreme weather conditions may greatly increase the likelihood of population extinction, which may be more frequent in the near future (Kingsolver et al. 2011).

Frampton et al. (2000) assessed the effects of spring drought and irrigation on farmland arthropods in the southern UK, finding that extreme weather conditions negatively affected the abundance of herbivores, mycophages, omnivores, and predators, suggesting potential implications for insectivorous wildlife. In a review of moth population declines in the UK, Fox (2013) cited habitat degradation (as a result of agricultural intensification and forestry practices) and climate change as likely drivers. The author concurrently indicated that there was little direct causal evidence for habitat loss, degradation, or fragmentation regarding moth declines rather “considerable circumstantial evidence.” Regarding chemical or light pollution, non-native species, or direct exploitation, Fox (2013) concluded that little evidence of negative population-level impacts currently exists. Ewald et al. (2015) evaluated the effects of long-term changes in climate (temperature and rainfall) and pesticide use on invertebrate abundance using a 42-year monitoring dataset from cereal fields in the southern UK. Eleven of 26 invertebrate groups demonstrated sensitivity to extreme weather events (hot/dry or cold/wet years), though most groups usually returned to long-term trends within a year (Ewald et al. 2015). Other orders showed no consistent patterns or increased over the study period. Although climate change correlated with some long-term trends in invertebrate abundance, pesticide use was considered more important in explaining the trends by these authors. However, as described by Leather (2018), cereal fields are intensively managed unnatural habitats receiving a number of inputs, including pesticides, and thus, likely not representative of the most biodiverse habitats.

By comparison, population dynamics of pest insect populations may actually increase in agro-ecosystems as a result of climate change. Implications of global climate change on food production resultant from changes in pest insect range, overwintering capacity, population growth, insect–host plant interactions, arthropod diversity, synchrony with crop hosts, green bridge host introduction, resistance development, increased risk of migrant pest invasion, etc., have been identified (Sharma and Prabhakar 2014). Losses due to insect pests, as well as changes in arthropod diversity and abundance, are forecasted to impact both crop production and food security (Sharma and Prabhakar 2014). Currently, it is estimated that pest insects consume the amount of food sufficient to feed more than 1 billion people, and with the global population projected to increase by 3 billion by 2050, it is likely that pest insects will increase in numbers and types during this timescale (Sharma and Prabhakar 2014), contrary to general insect trends.

With respect to land use change, an increasing number of studies have been implicating urbanization as a major factor contributing to globally declining insect numbers (Leather 2018). Although negative effects are not ubiquitous, urbanization is generally viewed as having a detrimental effect on invertebrate diversity and abundance (Jones and Leather 2012). Dennis et al. (2017) explored differences in rural versus urban indices for UK butterflies and concluded that national declines were more strongly associated with urban areas for 25 out of 28 species. Moreover, extrapolating vehicular-induced mortality results for pollinators along a section of highway in Ontario, Canada, across a number of landscape scales, Baxter-Gilbert et al. (2015) projected the potential for loss of hundreds of billions of Lepidopterans, Hymenopterans, and pollinating Dipterans each summer in North America alone. Thus, the potential direct implications of urbanization for insect populations are multifaceted and include habitat alteration (e.g., road and building construction), increased light pollution, and increased vehicle density, as well as indirect implications from increased resource demand from agriculture, commerce, fuel, and material.

Therefore, the hypothesis that pesticides, particularly neonicotinoids, are indirectly affecting bird population status via reductions in food resources should be considered with caution and within context of other more likely causes. Although ecologically plausible, the potential impact of crop protection products relative to other factors should be considered comparatively and holistically. In the USA, agricultural land use has declined since the 1980s, yet, as indicated in Table 2, honey bee toxicity by insecticide class has not changed considerably by generation since the 1950s, though avian toxicity (particularly chronic toxicity) and environmental fate have improved dramatically (i.e., less persistent and less toxic). While it would be disingenuous to suggest that insecticides do not pose any potential risk to non-target arthropods proximal to insecticide use, and thus indirectly to avian species, it would also be misleading to suggest that these compounds are the primary driver for such indirect effects.

## Discussion

Historically, declines in bird populations have been attributed to agricultural activity in US States (Carson 1962). It is worth noting that, at present, total cropland use as a fraction of total land area is consistent with that initially reported in 1910 at ~ 330 million acres, though agricultural land use peaked at ~ 390 million acres as recently as the 1980s (USDA ERS 2018). Since this peak in agricultural land use, the total acreage used specifically for cropping has decreased, as have the persistence and application rates of pesticides. And although some correlative studies have suggested pesticide use as a potential driver of farmland bird declines (Stanton et al. 2018; Mineau and Whiteside 2013; Chiron et al. 2014), others have concluded that declines in bird populations related to agricultural intensification are most closely associated with habitat loss (Hill et al. 2014; Belden et al. 2018). The results of the analyses conducted in the current study indicate fewer pesticide-related incidents, as well as more species with stable or increasing populations, in the period from 2005 to 2015 compared to 1960 to 2015, possibly as a result of agricultural chemicals with greater pest specificity and less persistence. Continued efforts to improve the agronomic and toxicological profiles of pesticides will be important for ensuring habitat viability as high-yield agriculture comparatively requires less land for production (Balmford et al. 2018). Technological advances in crop protection that pose the least possible relative impact on the environment, while still enabling food security for national and global consumers, should translate into positive effects on birds and biodiversity in general.

There are many opportunities to continue to explore sustainable approaches to intensified agriculture that could also be more supportive of healthy bird populations. For example, Sutton et al. (2017) recommended that the following be applied on at least 5–7% of arable land: (1) Field margins sown with wildflowers and pollen/nectar-rich species; (2) winter bird food via seed-rich habitats; (3) hedges cut incrementally or on 3-year rotation; and (4) ecological focus areas left uncropped and representing at least 3–5% of the landscape. Colhoun et al. (2017) and Walker et al. (2018) also found that targeted agri-environment schemes could have significant positive effects in priority bird species for conservation.

## Conclusions

With respect to bird species diversity and population viability in the USA, agricultural land and chemical use is analogous to a chapter in a much broader book that needs to be thoroughly read and considered holistically. Urban and suburban development has increased steadily over time, increasing direct interactions between humans (or the human-built landscape) and birds, as well as introducing new predators where they may not have existed in high density before (i.e., cats). Development has also meant habitat loss and fragmentation, as well as growing and shifting demands from human consumers whose preferences drive the agricultural market and economics of food and textile production. As stated previously, rigorous, science-based regulation of agricultural pesticides is essential and must continue; however, crop protection tools are also essential for ensuring food security, producing more (food) from less (land), and consequently enabling fidelity of habitat integrity and availability for the future. Focusing solely on the agricultural/chemical story within the broader biodiversity narrative lacks context and perspective. Certainly, the potential risks associated with pesticide use (including neonicotinoids) to birds and other taxa need to be thoroughly and rigorously evaluated, and they are, regularly and conservatively, by federal regulatory authorities. Relatively speaking, pesticides are not ranked as a significant contributor to global bird declines compared with other drivers (e.g., cats, buildings, automobiles), though that is not a justification for complacency and there is more that can be done to ensure sustainable global food production. In summary, the discussion requires more thorough, candid, and cooperative dialogue as the balance between human population growth and conservation of biodiversity is delicate, and we all have a role to play.

## Electronic supplementary material

Table S1 (DOCX 15 kb)
