# Supplementary material for: The agro-enabled urban revolution, pesticides, politics, and popular culture: a case study of land use, birds, and insecticides in the USA
Source: Environ Sci Pollut Res Int. 2019 May 26;26(21):21717–35. doi: 10.1007/s11356-019-05305-9 (PMC6647523; doi:10.1007/s11356-019-05305-9)
Supplement: Supplementary file 1 — (DOCX 15 kb) [file 11356_2019_5305_MOESM1_ESM.docx]

**The Agro-Enabled Urban Revolution, Pesticides, Politics, and Popular Culture:**

**A Case Study of Land Use, Birds, and Insecticides in the United States**

**Richard A. Brain^a*^, Julie C. Anderson^b^**

**^a^Syngenta Crop Protection LLC, Greensboro, North Carolina, USA**

**^b^Independent Consultant, Winnipeg, Manitoba, Canada**

**Supplementary Material**

Table S1: Life history details for bird species with the strongest declining trends in population according to the Breeding Bird Survey data collected between 1966 and 2015 and analyzed by Sauer et al. (2017). (Source: Cornell Lab of Ornithology’s Birds of North America - Rodewald 2015)

| **Common Name** | **Scientific Name** | **Habitat** | **Diet** | **Nesting** | **Migration** | **Range** | **Conservation Status** | **Notes** |
| --- | --- | --- | --- | --- | --- | --- | --- | --- |
| Blackpoll Warbler | *Setophaga striata* | Forest | Insects, some fruit | Open cup, mid-story | Long distance migrant | Breeding: Northern Canada  Migration: Mountain, Northern Plains, Lake States, Corn Belt, Appalachia, Northeast, Southeast, Southern Plains, Delta States | Common, but in steep decline | Long migrations (8000 km), remote habitats; direct collisions, exposure, and predation are major causes of mortality. |
| King Rail | *Rallus elegans* | Marshes | Crustaceans, aquatic insects | Ground | Resident to medium-distance migrant | Breeding: Southern Plains, Appalachia, Northeast, Delta States, Corn Belt  Year-round: Southern Plains, Southeast, Appalachia, Delta States | Declining, on yellow watch list | Sensitive to changes in hydrological regime; threatened by loss of wetlands; pesticides and vehicles also potential contributors to mortality. |
| Allen's Hummingbird | *Selasphorus sasin* | Open woodlands | Nectar, insects | Low to mid-story | Resident to medium-distance migrant | Breeding: Pacific  Migration: Pacific, Mountain  Winter: Mexico  Year-round: Pacific | Declining, on yellow watch list | Food availability a key driver for mortality; BNA notes BBS methodology not well-suited to hummingbirds. |
| Black Swift | *Cypseloides niger* | Forest | Insects | Open cup, high ledges | Medium to long-distance migrant | Breeding: Western Canada, Mexico  Migration: Pacific | Declining, on yellow watch list | Not well studied; very specific nesting conditions and low reproduction. |
| House Sparrow | *Passer domesticus* | Urban | Grains, seeds, insects | Cavity | Resident | Year-round: All U.S. regions | Low concern | Highest densities associated with livestock barns; decline could be tied to changing agricultural practice and increased efficiencies and/or lower numbers of livestock barns. |
| Northern Bobwhite | *Colinus virginianus* | Grasslands | Seeds, plants, insects | Ground | Mostly resident | Year-round: Southern Plains, Delta States, Appalachia, Southeast, Northern Plains, Mountain, Lake States, Corn Belt, Northeast | Common, but in steep decline | High annual mortality rates and reproductive rates; popular hunted species; feeding in agricultural areas with high potential for direct or indirect effects; losses primarily attributed to habitat loss via land use change. |
| Pinyon Jay | *Gymnorhinus cyanocephalus* | Open woodlands | Omnivore, esp. pine seeds | Mid-story to canopy | Resident | Year-round: Mountain, Pacific, Northern Plains | Declining, on yellow watch list | Over 60 years of federal and state programs to convert pine forests to pasture land, reducing habitat; highest mortality during/after breeding season (high rates of injury) and when food is either abundantly available (attracts predators) or unavailable (starvation). |
| Bendire's Thrasher | *Toxostoma bendirei* | Deserts | Insects, some plants | Open cup | Short to medium-distance migrant | Breeding: Pacific, Mountain  Year-round: Mountain, Mexico | Species of Special Concern in CA; on red watch list | Data patchy for BBS. |
| Chestnut-collared Longspur | *Calcarius ornatus* | Grasslands | Grains, seeds, grasses | Ground | Medium to long distance-migrant | Breeding: Canada, Mountain, Northern Plains  Migration: Mountain, Northern Plains  Non-breeding: Southern Plains, Mountain, Mexico | Declining, on yellow watch list | Endemic to plains; loss of native prairie has caused declines; likes disturbed/grazed areas i.e., as by grazing bison historically. |
| Bachman's Sparrow | *Peucaea aestivalis* | Open woodlands | Seeds, insects | Ground | Resident to short-distance migrant | Breeding: Southern Plains, Southeast, Delta States, Appalachia, Corn Belt  Year-round: Southern Plains, Southeast, Delta States, Appalachia | Declining, on red watch list | Prefers mature pine forests and open forest habitats; range has grown and contracted in response to land use trends; small number recorded during survey so hard to make firm assessment of trends. |
